# Evaluation of a group-based behavioural intervention (PROGROUP) versus usual care for weight management in adults with severe obesity: protocol for a randomised controlled trial with cost-consequence analysis and primary care implementation study

**DOI:** 10.1136/bmjopen-2025-115791

**Published:** 2026-07-24

**Authors:** Anton Barnett, Joanne Hosking, Mark Tarrant, Siobhan Creanor, Anne Spencer, Wendy Ingram, Jeanette Sanders, Dawn Swancutt, Jenny Lloyd, Rod Sheaff, Sarah Dean, Richard Byng, Lily Hawkins, Laura Hollands, Shokraneh Moghadam, Hanna Abraham, Paigan J Aspinall, Sarah Baldrey, Liz Edwards-Smith, Lucy Evans, Laura Gill, Hannah Gott, Sarah Hind, Mary O’kane, Emma O’shaughnessy, Steve Perry, Weiming Sun, Adrian Taylor, John Wilding, Lucy Wilson, Jonathan Pinkney

**Affiliations:** 1 Faculty of Health, University of Plymouth, Plymouth, UK; 2 Faculty of Health and Life Sciences, University of Exeter Medical School, Exeter, UK; 3 Occupational Health and Wellbeing Service, West Glasgow Ambulatory Care Hospital, Glasgow, UK; 4 University of Bristol, Bristol, UK; 5 Cumberland Centre, Damerel Close, Livewell Southwest, Plymouth, UK; 6 St James’s University Hospital, Leeds Teaching Hospitals NHS Trust, Leeds, UK; 7 Independent PPI member, Plymouth, UK; 8 Department of Cardiovascular and Metabolic Medicine, Clinical Sciences Centre, Aintree University Hospital, University of Liverpool, Liverpool, UK

**Keywords:** Obesity, Behavior, Clinical Trial, HEALTH ECONOMICS, Primary Care

## Abstract

**Introduction:**

Approximately 15 million people in the UK live with obesity and at least 5 million of these are people with severe obesity (PWSO). Severe obesity significantly compromises health, well-being and quality of life and reduces life expectancy. These adverse outcomes are prevented or ameliorated by weight loss, for which sustained behavioural change is the cornerstone of treatment. Several studies suggest the potential of group-based intervention in Specialist Weight Management Services (SWMS), but PWSO remain underrepresented in research, and evidence on optimal design and outcomes is limited. The success of the PROGROUP feasibility randomised controlled trial (RCT) (ISRCTN22088800) informed the development of this study and additional adjustments have been made in response to the rollout of obesity management medication in the National Health Service (NHS). This study aims to assess the effectiveness and cost-effectiveness of PROGROUP in SWMS and primary care.

**Methods and analysis:**

The RCT will be conducted in SWMS, alongside an implementation study in primary care. The RCT will recruit cohorts of 30 participants to be randomised 1:1 within-cohort to PROGROUP (intervention) or usual care (control). The implementation arm will recruit cohorts of 15 to PROGROUP. The primary objectives for the study are to undertake a process evaluation and economic evaluation of PROGROUP in the RCT and to assess its implementation in primary care. Baseline data and outcome differences at 6 months will be analysed. These will include weight, other clinical measures and patient-reported outcomes, including social and life-satisfaction measures.

**Ethics and dissemination:**

This study is approved by an NHS Research Ethics Committee (REC reference: 23/WS/0101). Results will be reported in a manuscript that will be submitted to a peer-reviewed medical journal as open access. A lay summary of the findings will be published online, and participants and sites will be signposted to this.

**Trial registration number:**

ISRCTN13721429.

**STRENGTHS AND LIMITATIONS OF THIS STUDY**

- The intervention design and trial are evidence-based, underpinned by social identity theory, and have involved co-design with patients and healthcare professionals.
- Following a successful feasibility trial, confirming feasibility of a full RCT, the study comprises a rigorously designed cluster randomised controlled trial with cost-consequence analysis and a single-arm primary care implementation study running in parallel.
- The study tests the PROGROUP intervention in two key settings: Specialist Weight Management Services and primary care.
- The inclusion of a cost-consequence analysis could provide insight into resource implications for the National Health Service (NHS).
- The contemporaneous changes in the licensing and rollout of new obesity management medications by the NHS led to under-recruitment to the study, which may limit some outputs of the definitive trial.

## Background and rationale

### The challenge of treating severe obesity

 Approximately 15 million people in the UK live with obesity and at least 5 million of these live with severe obesity (Body Mass Index (BMI)≥35 kg/m^2^).^1^ At higher levels of BMI (eg, 40–45 kg/m^2^), which is commonly seen in UK National Health Service (NHS) specialist weight management services (SWMS), average loss of life expectancy is 8–10 years,^2^ meaning that approximately 1.5 million adults in the UK face early death attributable to this condition—and meanwhile, living with substantially compromised psychosocial health, well-being and quality of life.^3 4^ With recent estimates predicting a dramatic rise in obesity prevalence, the All Party Parliamentary Group describes obesity as ‘a problem the country cannot afford to defer to the next generation’.^5^

Currently, the annual cost of obesity to the NHS is estimated to be between £2.47 billion^6^ and £5.1 billion,^7^ and this is predicted to rise to £10 billion by 2050. The wider costs to society are even greater—estimated at £50 billion per year.^8^ Societal impacts are felt through higher levels of worker absenteeism, reducing productivity,^9^ increased dependence on benefits, early retirement, increased levels of chronic disease^10^ and mental health concern^11^ arising from obesity-related experiences such as weight stigma.^6^ Although all sections of society are subject to obesity, those of lower socioeconomic status, as well as ethnic minorities, experience a greater burden.^7^

The World Obesity Federation highlighted the global impact of obesity and how most of its detrimental effects are prevented by weight loss.^12^ However, severe obesity is a complex condition that is influenced by psychological, social and environmental factors, including childhood trauma, stigma and mental health problems, socioeconomic status and availability (and marketing) of unhealthy foods.^8 13^ The foundation of the non-surgical treatment of severe obesity is sustained behavioural intervention, centred on modifications to diet, physical activity and lifestyle. The recent advent of the new generation of incretin-based drugs will offer eligible individuals additionally improved prospects for weight loss.^14 15^ The NHS in England commissions weight management services, yet the most effective and cost-effective ways to structure and deliver foundational behavioural weight loss interventions for people with severe obesity (PWSO), with or without accompanying drug therapies, are poorly evidenced. Similarly, there is a need to establish behavioural frameworks that optimise and maintain long-term benefits for those who receive drug treatments. The scale and cost of severe obesity are such that there is a need to identify the most effective and cost-effective approaches to delivering the core behavioural intervention.

### Group-based behavioural interventions

The potential of group interventions to support treatment of long-term health conditions is recognised.^16 17^ Key psychosocial resources linked to behaviour change, including social support and self-efficacy,^18^ can be triggered when group members form a sense of social connectedness or shared social identity, with other group members.^19 20^ Being socially connected to others is associated with improvement in a variety of health outcomes, including reduction in mortality rate.^21^ Indeed, social connectedness appears to have a stronger influence on mortality than excessive alcohol consumption and smoking.^22^ A recent systematic review and meta-analysis confirmed the positive impact of interventions which specifically build shared social identity on a range of health outcomes, including psychosocial, cognitive, mental and physical health. Overall effects indicated a moderate-to-strong impact on health (standardised effect size of 0.66).^20^ Group interventions are increasingly used to deliver behaviour change in healthcare, but there is a need for more UK-centred evaluation and clearer delineation of group delivery components.^16 17^ There are additional, practical reasons to consider adopting group-based interventions; the scale of severe obesity in the population, and the rapidly rising demand for treatment, including with new drugs, poses considerable challenges for conventional delivery models.

### Group-based behavioural interventions in the setting of specialist weight management services

Group interventions may add value to services for PWSO by contributing health benefits beyond pragmatic delivery of programme content.^16 17 23–25^ In our evaluation of a UK group-based SWMS, patients reported how the shared social identity formed within the treatment setting was a key mechanism structuring their engagement with and progression through the group programme.^26^ Consistent with this observation, our review of Randomised Controlled Trials (RCTs) of group-based behavioural interventions reported superior weight loss at 6 months in group interventions versus controls (mean weight loss difference=−3.6 kg).^27^ Differences persisted, although slightly reduced up to 18 months.^28^ Findings indicate benefits of group*-*based programmes, but mean (baseline) BMI in the RCTs included was only 33.6 kg/m^2^ and so generalisability to PWSO is unclear. The ‘Look AHEAD’ trial in the USA also included a group-based intervention for weight loss (mean baseline BMI=36 kg/m^2^),^29^ but it is uncertain whether findings extrapolate to the UK and to PWSO, as opposed to those with diabetes.

The development of group processes, and shared social identity specifically, is not currently considered in the design of SWMS group programmes and is consequently neglected in practice.^17^ Without active facilitation, group processes may cause interpersonal conflicts or encourage the formation of group norms and cognitions that undermine behaviour change techniques^30^ that might otherwise be effective.^31^ Our recent research provides an evidence base for understanding how to assemble groups in clinical settings and capitalise on their therapeutic potential,^25 32 33^ not least as we enter a new era of drug treatment, but there is a need for more UK-centred evaluation of group delivery in SWMS.^17 34^ In summary, several studies suggest the potential of group-based intervention in SWMS. However, PWSO are inadequately represented in research, and previous studies pre-dated new evidence on best practice for developing group-based interventions.^25 35 36^ Therefore, it remains uncertain whether the adoption of group-based intervention in SWMS would enhance patient outcomes and be cost-effective.

The PROGROUP research programme aims to address this challenge, by establishing the evidence needed for the successful implementation of a new group-based behavioural intervention (PROGROUP) for PWSO in SWMS settings. A feasibility RCT^37^ met its prespecified progression criteria, indicating that a definitive trial was feasible, while also informing several design modifications for the definitive trial (ISRCTN22088800). The findings of the feasibility trial and the prior work package within the research programme informed the design of a definitive RCT and enabled optimisation of outcome measure selection and frequency of assessment, as well as optimisation of the intervention itself. The definitive trial design was: ‘a multi-centre, two-arm, individually randomised controlled, partially clustered, assessor-blinded, adaptive superiority trial of PROGROUP (group-based intervention) versus usual care (control) with parallel process evaluation and health economic evaluation and an internal pilot, taking place in the Specialist Weight Management Services of up to 15 NHS secondary care trusts’.

### Changes to the definitive trial protocol during the trial

During the recruitment phase of the definitive trial, in response to the incidence of elective bariatric surgery and obesity management medication (OMM) use in the trial population (these being exclusion criteria at consent), the primary endpoint was changed from 12 to 6 months post-randomisation. Accordingly, sample size re-estimation was warranted, and the sample size reduced from 990 to 780.

Thereafter (during the recruitment phase), there were increasing challenges at site-level (eg, NHS commissioning uncertainty for SWMS; introduction of OMMs) and patient-level (eg, expectation of OMMs; inability to commit to attend sessions due to travel costs or time). When assessed against pre-defined progression criteria for recruitment and completion of baseline data collection, it was deemed that the definitive, statistically powered trial was no longer feasible. In consultation with the independent trial steering committee (TSC), sponsor and funder, an adjusted programme of work was agreed that maximises data collection while remaining relevant to the original objectives of the research programme and addresses the complexities and realities of current practice.

The aims of the adjusted programme of work are:

- Examine whether PROGROUP offers a cost-saving way of delivering group-based care, via a cost-consequences analysis.
- Examine how best to implement PROGROUP in the NHS primary care setting to extend the logic model to new context.

Changes to the original protocol are as follows:

1. Recruitment of participants: use of OMM will be permissible from the outset, if deemed clinically appropriate; in the original trial design, use of OMMs was an exclusion criterion.
2. Retention of participants: there is greater flexibility to deliver certain PROGROUP sessions online.
3. Final follow-up of participants: will be conducted at 6 months post-randomisation; the 12-month time-point is omitted.
4. Data analysis:
  - Statistical analysis will be of a descriptive nature only; the original aim was to demonstrate superiority of PROGROUP (intervention) compared with usual care (UC) (control) with respect to weight change between baseline and 12 months post-randomisation in adults with severe obesity.
  - The economic evaluation will focus on a cost consequences analysis for the two trial arms; the original economic evaluation objectives included plans for a ‘within-trial’ cost-effectiveness analysis of PROGROUP versus UC and a ‘lifetime’ analysis to estimate the lifetime cost-effectiveness of weight loss.
5. A single-arm implementation study of PROGROUP delivery in the primary care setting is introduced.

This protocol describes the adjusted programme of work.

### Aims

This study aims to: (1) examine, via a cost-consequences analysis, whether a new group-based behavioural intervention (PROGROUP) for PWSO in SWMS settings offers a cost-saving way of delivering group-based care; (2) examine how best to implement PROGROUP in the NHS primary care setting to extend the logic model to new context.

### Overall study design

The study is a multi-centre, two-arm, individually randomised controlled, partially clustered, assessor-blinded trial of PROGROUP (group-based intervention) versus UC (control) for PWSO, with descriptive statistical analysis, cost-consequence analysis and parallel process evaluation in the secondary care setting, and an implementation study in primary care.

The study protocol conforms with the Standard Protocol Items: Recommendations for Interventional Trials (SPIRIT) reporting requirements.^38^

### Participants

Participants are adults with severe obesity.

### Objectives

The primary objective is change in weight (kg) from baseline to 6 months post-randomisation to be analysed descriptively; no formal statistical hypothesis testing will be undertaken. The estimand is defined as:

- Population: adults with severe obesity (as defined by trial inclusion/exclusion criteria).
- Endpoint: change in weight between baseline and 6 months post-randomisation.
- Treatment condition: PROGROUP intervention compared with UC.
- Key intercurrent events: discontinuation of the PROGROUP intervention for any reason; the initiation of weight loss drug therapy or surgery—these are addressed in the treatment condition and handled with the treatment policy strategy.
- Population-level summary: difference in mean weight change from baseline to 6 months post-randomisation between treatment conditions.

Process evaluation objectives are as follows:

- Assess fidelity to delivery and mechanisms of action of PROGROUP.
- Ascertain facilitator and participant engagement with PROGROUP.
- Ascertain differential uptake and retention by gender, socioeconomic status and ethnicity.
- Document any contamination effects across the trial arms.
- Document organisational and policy contexts at each site affecting implementation of PROGROUP.
- Document patterns of variation in the structure and delivery of UC between sites.
- Ascertain the key issues that hindered the trial progress from the patient and service perspective.

Economic evaluation objectives are as follows:

- Refine estimates of the intervention costs.
- Determine whether PROGROUP produces cost savings.
- Explore the costs and outcomes separately for those taking OMM and those who are not.
- Update and validate the Health Economics simulation model from the feasibility RCT in order that this may become a template for modelling severe obesity.
- Explore the extent to which outcomes are correlated for SWMS, which has the potential to inform power calculations in future studies for SWMS.

Primary care implementation study objectives are as follows:

- Determine how delivery of PROGROUP is achieved in practice.
- Explore which factors impede delivery of PROGROUP and which factors facilitate it.
- Ascertain how access, uptake and engagement of the PROGROUP intervention varies across key patient demographics including ethnicity and socioeconomic status.
- Ascertain what training adaptations are required for primary care staff who deliver the intervention.
- Explore how the intervention fits alongside the NHS model for prescribing OMM.
- Explore how PROGROUP might shape patients’ expectations and motivations for behavioural change in relation to (potential) OMM.
- Explore how the patient experience of PROGROUP might differ across SWMS and primary care settings.
- Determine how the delivery and costs of PROGROUP differ across SWMS and primary care settings.

### Outcomes

#### Primary outcome

The primary outcome for the RCT is the change in weight (kg) from baseline to 6 months post-randomisation.

#### Secondary outcomes

Change in the following between baseline and 6 months post-randomisation:

- The percentage of participants achieving ≥5% wt loss (minimum clinically worthwhile weight loss) and ≥10% wt loss.
- Physiological measures
  - BMI derived from height at baseline and weight at the time-point (kg/m^2^).
  - HbA1c (mmol/mol).
  - Systolic blood pressure (mmHg).
  - Total cholesterol (mmol/L).
  - HDL cholesterol (mmol/L).
  - Triglycerides (mmol/L).
- Co-morbidities and medication use.
- Validated participant self-reported outcome measures:
  - Alcohol consumption (Alcohol Use Disorders Identification Test, AUDIT-C^39^).
  - Eating behaviour (Adult Eating Behaviour Questionnaire^40^).
  - Physical activity (International Physical Activity Questionnaire^41^).
  - Quality of life (EQ-5D-5L^42^).
  - Well-being (ICEpop CAPability measure for Adults, ICECAP-A,^43^ Patient Health Questionnaire-4, Patient Health Questionnaire-4,^44^ Self-esteem,^45^ Life satisfaction,^46^ Loneliness^47^).
  - Social identification.^48^
- Participant self-reported health, social care and wider societal resource use (ie, use of primary care and community-based services, hospital services, pharmacological interventions for weight loss, support from others, self-funded weight loss interventions and employment status) also reported at 3 months post-randomisation to facilitate participants’ recall.

For participants recruited prior to implementation of the adjusted programme of work, the above outcomes will have been collected at 12 months post-randomisation, and these will be reported.

#### Primary care implementation study outcomes

The implementation study will assess the:

- Recruitment rate and screening conversion rate.
- Reasons for patients declining to participate.
- Intervention attendance rate and intervention engagement.
- Assessment of the mechanisms of action of the PROGROUP intervention.
- Intervention fidelity assessment.

## Randomised controlled trial

### Recruitment and informed consent

**Figure 1:**
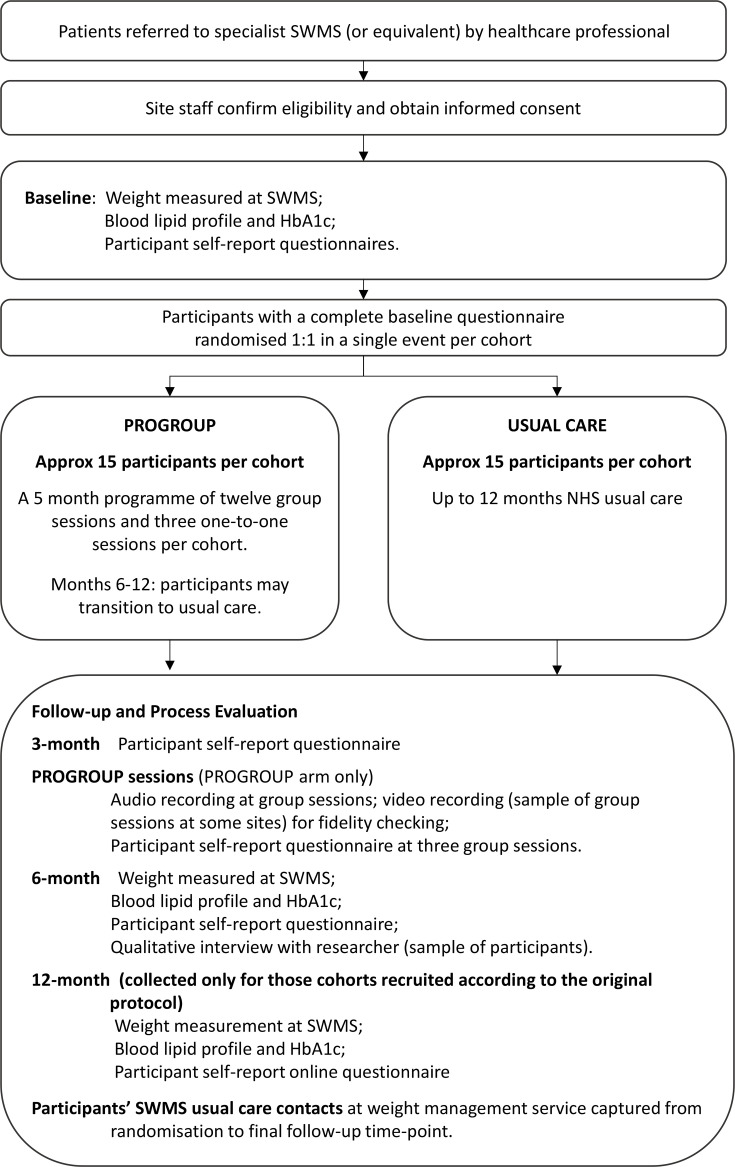
Summary flow chart for the randomised controlled trial element of the PROGROUP study. NHS, National Health Service; SWMS, Specialist Weight Management Services.

The group-based nature of PROGROUP necessitates the confirmed recruitment and participation of a sufficient number of participants within a recruiting site prior to randomisation. As such, each site will aim to recruit pools of ~30 participants (but with flexibility to recruit 24–36) to be randomised at a single time-point as a cohort. A summary of participants’ flow in the RCT study is shown in figure 1.

Potential participants will be identified from patients who have been referred to SWMS. Staff will provide potential participants with a localised copy of the Study Summary Leaflet and Participant Information Sheet either by post or email prior to their initial appointment, or in person at this appointment. Eligibility confirmation will be undertaken by members of the site staff. All participants will be given information about the nature and objectives of the study and possible risks associated with their participation. Informed consent will be obtained from all participants prior to randomisation and after at least 24 hours have elapsed since they receive study information to allow the opportunity to ask further questions about the study. The first participant was consented on 31 May 2024 and the participants in the first cohort were randomised on 23 July 2024. Recruitment will end in February 2026.

### Randomisation and allocation concealment

Participants will be randomised 1:1 into PROGROUP or UC using block simultaneous randomisation on a web-based randomisation system provided by the Clinical Trials Unit (CTU). Allocations will be assigned in order of the participants’ baseline questionnaire completion dates by a data manager in the CTU (using a static list created by a statistician who is not part of the trial team).

### Blinding

Due to the nature of the intervention, participants will not be blinded. Outcome assessors conducting follow-up will be blinded to treatment allocation. Success of outcome assessor blinding will be evaluated by asking assessors to record the treatment group they think a participant has been allocated to the follow-up visit. The trial statisticians undertaking the statistical analysis will be blinded for the primary analysis. Unblinded data will be made available to the Steering Committee on their request.

### Treatments

#### Usual care

UC will be an existing programme consistent with the principles of the National Institute for Health and Care Research (NICE) guidelines CG189^49^ and NG246.^50^ It is expected that there will be some variation in the structure and delivery of UC, reflecting normal practice nationally.^51^ Group education activities that are sometimes used in UC (eg, introduction meetings, healthy eating education, activity sessions, bariatric education) will be permitted. However, dedicated group activities beyond provision of didactic educational information, such as group discussions and activities including group-based problem solving and consensus forming, which is the defining feature of the PROGROUP intervention and is not present in the great majority of SWMS UC interventions, are not permitted in this study.

In this study, for a given cohort of participants, the UC programme will commence at the same time as the PROGROUP programme.

#### PROGROUP

PROGROUP is a manualised intervention and will be provided to participants in accordance with the manual by purposely trained facilitators from a multidisciplinary team (typically, nurses, dieticians or physiotherapists) at each site.

Participants will be asked to make every reasonable effort to adhere to the intervention programme schedule. The importance of engagement with all trial activity will be emphasised to all participants in both arms the PROGROUP intervention specifically via a study-specific charter.

The PROGROUP intervention consists of 15 sessions over 5 months:

- Weeks 1–2: Each participant will attend an initial one-to-one meeting with a facilitator, to review medical history, motivations and preparations for joining the group.
- Weeks 3–10: Participants will attend eight consecutive weekly group sessions, to teach and build on behavioural skills.
- Weeks 11–12: Each participant will attend an interim one-to-one meeting with the facilitator to review progress.
- Weeks 14–18: Participants will attend three consecutive fortnightly group sessions focusing on behavioural maintenance.
- Weeks 19–20: Final one-to-one meeting with the facilitator, for clinical review to develop plans for individualised continuation of programme learning.
- Week 22: Final group session to conclude intervention and prepare for self-directed implementation of programme learning.

A PROGROUP group will consist of approximately 15 participants (anticipated range of 12–18 participants). Each PROGROUP group session will be face-to-face and will take 2 hours inclusive of breaks. Group sessions will be delivered face-to-face, although for those recruited after the final follow-up was changed to 6 months, sites may opt to deliver some/all of the group sessions 4–8 remotely. These sessions will be audio recorded to be analysed using inductive thematic analysis, with video recordings obtained from a sample of sites. The one-to-one meetings will be 1 hour long and can be held either face-to-face or remotely (eg, telephone, video call) to offer flexibility to both participants and facilitators.

While there is no established ‘minimum dose’ for PROGROUP, all participants (in both arms) will be asked to make every reasonable effort to adhere to their programme schedule.

### Eligibility

#### Site eligibility

The UC programme at a site must meet the stated criteria, have a sufficient referral rate (or waiting list size) to be able to recruit patients within the study period, and have a multidisciplinary SWMS team with the capacity to run the PROGROUP sessions.

#### Inclusion criteria

Participants must satisfy all the following:

1. Registered with the SWMS (in the UK).
2. A BMI≥37.5 kg/m^2^ or BMI≥32.5 kg/m^2^ with at least one significant comorbidity* at the point of consent for individuals with a South Asian, Chinese, other Asian, Middle Eastern, Black African or African-Caribbean family background. A BMI≥40 kg/m^2^ or BMI≥35 kg/m^2^ with at least one significant comorbidity* at the point of consent for all other individuals.
3. Age≥18 years.
4. Willing to be randomised to either PROGROUP or UC.
5. Willing to have weight measured on at least three occasions: baseline and 6 months (and 12 months for those recruited before the final follow-up was changed to 6 months).
6. Willing to provide blood samples and blood pressure readings on three occasions: baseline and 6 months (and 12 months for those recruited before the final follow-up was changed to 6 months).
7. Considered suitable for group-based care.
8. Have capacity to consent.

* Type 2 diabetes, hypertension, dyslipidaemia, cardiovascular disease, osteoarthritis, obstructive sleep apnoea and ‘other’. Suitability of ‘other’ will be determined on a case-by-case basis.

#### Exclusion criteria

Participants meeting any of the following will be excluded:

1. Already undergone bariatric surgery.
2. Are scheduled, or have made their own plans, to undergo bariatric surgery during the trial.
3. Currently taking the following pharmacotherapy for the indication of weight loss: Semaglutide, Tirzepatide, Liraglutide, Orlistat or any off-licence weight-reducing pharmacotherapy such as the stimulant appetite suppressants phentermine and diethylpropion. Commencing these medications from post-randomisation is allowable. (For those recruited prior to implementing the adjusted programme of work, starting these medications within 6 months post randomisation was not allowable.)
4. Currently engaged in any other weight management trial.
5. Unwilling or unable to attend the SWMS for intervention/UC appointments.
6. Intending to relocate outside the geographical region during the trial period.
7. Participants who have significant difficulties in adequate understanding of English, or a sensory impairment, such that they are unable to sufficiently understand/access the trial documentation or engage in intervention/UC appointments, in the absence of a local provision of translated materials or communication aids.

### Collection of data

Data will be entered by site staff or participants, as applicable, to an electronic data capture system built and maintained by the CTU, comprising a bespoke web-based database and REDCap Community. See table 1.

**Table 1:** Study schedule

		Pre-baseline	Baseline	Randomisation	Post-randomisation		
Time point			*t_0_*		3 months (coincides with intervention mid-point)	6 months (−2 weeks to +4 weeks window) t_*1*_	12 months* (−2 weeks to +4 weeks window) *t*_*2*_
Enrolment:							
Eligibility screen		X	X				
Informed consent			X				
Randomisation				X			
Interventions:							
Control group:	Usual care (dotted line)		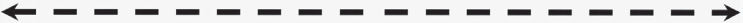				
Intervention group	Usual care (dotted line) PROGROUP (solid line)		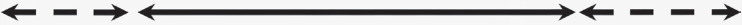				
Assessments:							
Demographics			X				
Weight (kg) within 6 weeks prior to randomisation			X			X	X
Height (cm) within 6 weeks prior to randomisation			X				
HbA1c (mmol/mol) at or within 1 month prior to baseline visit (eg, from medical records).			X			X	X
Lipid profile (mmol/L) at or within 1 month prior to baseline visit (eg, from medical records).			X			X	X
Systolic blood pressure (mm Hg)			X			X	X
Co-morbidities			X			X	X
Medications†			X			X	X
Adult Eating Behaviour Questionnaire (AEQB)‡			X			X	X
Alcohol use (Audit-C)‡			X			X	X
International Physical Activity Questionnaire‡ (IPAQ) short form‡			X			X	X
ICEpopCAPability measure for Adults (ICECAP-A)‡			X			X	X
Patient Health Questionnaire-4 (PHQ-4)‡			X			X	X
Self esteem and life satisfaction measure(s)‡			X			X	X
EQ-5D-5L questionnaire‡			X			X	X
Social identification measure‡			X		X	X	X
Loneliness measure‡			X			X	X
Resource use questionnaire‡			X		X (weight loss medication and surgical procedures only)	X	X
Participant travel costs (for intervention costing)‡					X	X	
Safety monitoring:							
Adverse event reporting				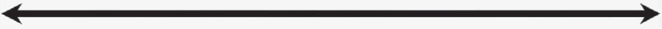			

* Applies only to participants randomised prior to implementation of the adjusted programme of work.

† Self-reported by participants and also reported by site staff with access to medical records.

‡ Self-reported by participants.

#### Baseline measurements

For eligible and consented participants, site staff will record baseline clinical measures: height, weight, blood lipid profile (total cholesterol, HDL cholesterol, triglycerides), systolic blood pressure and glycaemia measurement (HbA1c). Informed consent will be documented on a study-specific consent form (see online supplemental material 1).

Due to the group-based nature of the PROGROUP treatment, sites are required to recruit participants as a cohort (of 24–36 participants) prior to randomisation of that cohort.

To minimise the gap between baseline clinical measurements and randomisation, self-reported baseline assessment questionnaires will only be issued to participants after a full cohort has been recruited. The decision to declare a cohort complete will be made by the Co-Chief Investigators in consultation with the Trial Management Group.

The time from eligibility assessment (including weight measurement for derivation of BMI) to randomisation must be less than 6 weeks. In the event that this interval exceeds 6 weeks, baseline weight will be re-measured prior to proceeding with randomisation. Other clinical baseline measures will not be repeated since these are considered unlikely to change in this interval.

#### Follow-up assessments

Participants will attend a face-to-face clinic where, using a study-specific electronic case report form (eCRF), a blinded outcome assessor will record participant weight, HbA1c, lipid profile and blood pressure, and any use of OMM or bariatric surgery undertaken. Alternative arrangements for measurement of weight might be appropriate in exceptional circumstances and on a case-by-case basis, for example, weight measured at the participant’s home by trained staff using calibrated clinic weighing scales.

Separately, participants will complete the self-reported measures.

### Withdrawal

The risk of harm to participants caused by treatment in either trial arm is very low; however, there is a potential risk of participants requesting to withdraw from the PROGROUP arm for psychological reasons (eg, being uncomfortable with the group-based format of the PROGROUP arm, as observed in the feasibility RCT). Participants may choose to withdraw at any time.

Intervention facilitators may also choose, at their discretion, to withdraw participants from the PROGROUP programme. Grounds for withdrawing participants from the PROGROUP programme may include, for example, disruptive behaviour or wilful non-engagement.

Withdrawal from the PROGROUP programme, and the reason if provided, will be clearly documented in the participant’s clinical records reported using a study-specific eCRF.

All participants withdrawn from PROGROUP or UC will be encouraged to continue with follow-up visits and assessments as per the trial protocol.

Data collected prior to withdrawal from follow-up will be included in the study analysis. Participants who withdraw after their cohort has been randomised will not be replaced with new participants, due to the nature of the trial intervention.

### Adverse events

The likelihood of participants being harmed by either the PROGROUP intervention or any of the trial procedures is very low. As such, the collection and reporting of events in the PROGROUP trial is restricted to only those events which are deemed to be serious adverse events (SAEs).

The primary means of detecting SAEs will be at the follow-up clinic visits and the intervention sessions. At each contact point, participants will be asked to describe any adverse events (AEs) they have experienced.

### Sample size and statistical methods

The original definitive trial sample size calculation was informed by previous literature^52–57^ and the PROGROUP feasibility RCT. The recruitment target after changing the primary endpoint to 6 months was 780 participants, from~26 cohorts across eight recruiting sites. This target was calculated based on detecting the pre-specified between-group mean difference in weight loss of 3 kg, with 90% power and 5% two-sided significance level, with assumed SD of 7 kg, mean cluster size in each treatment arm (at 6 months) of 10 participants (with cluster size variability of 9), intra-cluster correlation coefficient of 0.025 in UC and 0.2 in PROGROUP, and allowing for 30% dropout rate.

Given that the RCT will be underpowered to detect the pre-specified between-arm difference in weight change under the original sample size assumptions, statistical analysis of the RCT will focus on a descriptive approach.

Descriptive statistics of participants’ demographic and baseline characteristics will be presented by allocated groups and overall. Continuous data will be reported with means and SD if they appear normally distributed, otherwise the median and inter-quartile range will be used. For categorical data, frequency and proportions will be presented.

The primary descriptive analysis of all outcomes will follow an intention-to-treat approach. For the primary estimand, the analysis will use the treatment policy strategy to handle the key intercurrent events of intervention discontinuation and initiation of weight loss medication. A mixed effects model will be fitted for the primary outcome, including participants with weight measured at baseline and at 6 months post-randomisation, allowing for differential clustering between the control and intervention groups, and adjusting for relevant individual covariates, site, and baseline weight. Model parameter estimates and CIs will be presented; however no formal hypothesis tests will be conducted. Missing data will not be imputed.

The number of participants achieving a clinically meaningful weight loss of ≥5% and ≥10% baseline body weight at 6 months post-randomisation will be reported as the number and proportion meeting this threshold by trial allocation arm and overall.

Sensitivity analyses will use subgroups of the full dataset that exclude participants that

- Discontinued from the treatment.
- Used OMM during the trial period.
- Underwent bariatric surgery.

If any sites offer group-based care as part of their UC, a subgroup analysis will be undertaken where the effect of UC type will be explored.

Full details of the planned analyses will be described in the statistical analysis plan (SAP), which will be finalised prior to database lock. Once finalised, the SAP will be uploaded to the ISRCTN registry along with the trial protocol.

A formal sample size calculation has not been conducted for the cost consequence analysis. For this analysis, we estimate that 120–140 participants across six cohorts is feasible, based on predictions for recruitment of sites and participant recruitment rate.

### Process evaluation

Data will be collected through interviews, which will be captured using a digital recorder, and transcribed. Each interview group (participants, facilitators, clinical lead and service managers) will be analysed using thematic analysis and the Framework approach^58^ to organise and code the data. The context-mechanism-outcome framework will be used as standard in realist evaluations,^59^ supplemented with any other themes emerging directly from the data during coding. Attention will be paid to negative, or deviant cases to inform developing themes and interpretation. The generated themes will be used to update the logic model.

Focus groups with facilitators will be conducted to further refine understanding of the PROGROUP mechanisms and wider experiences of the intervention. Interview and focus group data will be analysed thematically.

Any unintended consequences of the intervention will be captured and synthesised and fed into programme and training manuals as appropriate for future implementation.

While contamination between arms is unlikely, processes will be in place to minimise it. Facilitators will be asked whether they used aspects of PROGROUP when working with UC patients. The importance of preventing contamination will be covered in the training sessions and followed up in the weekly support calls.

Facilitators delivering PROGROUP will complete a fidelity-to-session content checklist at the end of each group session.

Intervention participants will complete a Group Processes Questionnaire at three time-points (beginning, middle and end of the intervention). This questionnaire will assess the following constructs:

- Social identification with the PROGROUP group.
- Social support derived from the PROGROUP group.
- Self-efficacy attributed to the PROGROUP group.
- Perceptions of the PROGROUP group facilitator(s).
- PROGROUP group atmosphere, including perceptions of group conflict.
- Social group membership beyond PROGROUP.

This questionnaire will include additional questions at the second and third data collection points to ascertain understanding of and enactment of behaviour change strategies introduced during the programme and continuity of the group makeup.

At 6 months, all participants (both trial arms) will be asked 6–8 questions about their contact with other participants receiving the SWMS support outside of their UC or PROGROUP sessions, any lifestyle changes made, anxiety about their weight, contamination effects and experience of receiving either UC or PROGROUP.

### Cost-consequence analysis

Information about the various costs and outcomes (consequences) will be presented, providing a detailed breakdown of both positive and negative impacts, an approach that has been used with stroke and other services.^60 61^

The outcomes to be considered include weight, EQ-5D-5L, ICECAP-A collected at baseline and 6 months, and important clinical factors (cholesterol, systolic blood pressure, HbA1c) required by the patient-level model, also collected at baseline and 6 months. Within this analysis, the patterns of costs, Quality Adjusted Life Years (QALYs, measured by EQ-5D-5L) and capability well-being (ICECAP-A) will be explored for those participants taking obesity management medication and those who are not to explore whether patients on obesity management drugs may experience a reduction in quality of life or well-being from AEs during drug treatment.^62^

### Data management

The CTU will oversee the management of data for the PROGROUP trial. Participant screening and outcome data will be entered into a bespoke web-based system, which stores all electronic data on Microsoft Azure servers. Clinical data will be entered into REDCap, a web-based data capture system, by authorised personnel from participating sites. Access to REDCap is regulated using usernames, encrypted passwords, and two-factor authentication. The system maintains an electronic audit trail and hosts in-built validation mechanisms to ensure data integrity and security, including validation checks at the point of data entry.

To ensure data quality and completeness, the CTU team will regularly monitor the data using validated R scripts. The monitoring process includes post-entry validation checks, ensuring completeness of critical data items, surveillance of safety and withdrawal reports and audit trail monitoring.

No direct identifiers will be shared with the trial statisticians, and access to directly identifiable information within the data capture system will be restricted to only users who require access.

Data from the PROGROUP trial will be retained and accessible for 5 years post-study completion. Participants may also consent to an optional clause to collect their NHS number to facilitate long-term access to relevant health data routinely collected by health services (eg, NHS England).

### Patient and public involvement

Patient and public involvement has contributed by a co-applicant with lived experience of weight management services and advocacy, the Patient Advisory Group, and via representation on the independent Steering Committee.

### End of trial definition

For cohorts recruited under the original protocol, participants will complete their involvement in the trial after approximately 12 months post-randomisation, at the 12 months follow-up assessment.

For cohorts recruited according to the adjusted plan, this will occur at 6 months post-randomisation.

The trial will end on completion of all final follow-up data collection.

## Implementation study

### Aim

The single-arm implementation study of the PROGROUP intervention in primary care will explore the conditions that support or impede the implementation of PROGROUP in NHS primary care for people living with severe obesity and its impact (positive and negative) on patients and healthcare professionals. These findings will be compared with delivery of PROGROUP and patient experience in secondary care. The intervention is as described for the RCT.

### Participants

#### Inclusion criteria

Patients must satisfy all of the following criteria to be enrolled in the study:

- Registered with the participating UK general practitioner (GP) practice.
- Meet the criteria for referral to the SWMS.
- A BMI≥37.5 kg/m^2^ or BMI≥32.5 kg/m^2^ with at least one significant comorbidity* at the point of consent for individuals with a South Asian, Chinese, other Asian, Middle Eastern, Black African, or African-Caribbean family background. A BMI≥40 kg/m^2^ or BMI≥35 kg/m^2^ with at least one significant comorbidity* at the point of consent for all other individuals.
- Aged≥18 years.
- Willing to have weight measured on at least two occasions (baseline and 6 months).
- Willing to provide blood samples and blood pressure readings on two occasions (baseline and 6 months).
- Considered suitable for group-based care.
- Have capacity to consent.

* Type 2 diabetes, hypertension, dyslipidaemia, cardiovascular disease, osteoarthritis, obstructive sleep apnoea and ‘other’. Suitability of ‘other’ will be determined on a case-by-case basis.

#### Exclusion criteria

Patients who meet any of the following criteria will be excluded from study participation:

- Already undergone bariatric surgery.
- Are scheduled, or have made their own plans, to undergo bariatric surgery during the course of the trial.

- Currently taking the following pharmacotherapy for the indication of weight loss: Glucagon-like peptide-1 (GLP-1) analogues (eg, Semaglutide, Tirzepatide, Liraglutide)**, Orlistat, or any off-licence weight-reducing pharmacotherapy such as the stimulant appetite suppressants phentermine and diethylpropion. Commencing these medications during the study is allowable.
- Currently engaged in any other weight management trial.
- Unwilling or unable to attend the locations for the PROGROUP sessions and study visits.
- Intending to relocate outside the geographical region during the trial period.
- Participants who have significant difficulties in adequate understanding of English, or a sensory impairment, such that they are unable to sufficiently understand/access the trial documentation or engage in the intervention sessions, in the absence of a local provision of translated materials or communication aids.

** Taking GLP-1 analogues for diabetes control (rather than weight management) for at least 12 months prior to screening is not an exclusion criterion.

### Recruitment and informed consent

Potential participants will be patients at the GP practice who are suitable for referral to a SWMS. Informed consent will be documented on a study-specific consent form (see online supplemental materia 1). Patients at the GP practice may also self-refer in response to information about the study on display at the GP practice.

### Sample size

A formal sample size calculation has not been conducted for the single-arm implementation study. The aim is to recruit three GP practices with 15 participants recruited at each site, totalling 45 participants.

### Data collection and analysis

Data collection and analysis will follow the approach taken for the RCT. The exception is that an end-of-session questionnaire, to be completed by each participant after each PROGROUP session, has been introduced.

Demographic and baseline clinical characteristics of the participants will be descriptively summarised, providing an overview of the study sample. Weight and BMI at baseline and 6 months will be reported. The participant self-reported measures at baseline, 3 and 6 months will be collected and analysed as described for the RCT.

Attendance data at PROGROUP sessions will be reported to understand the reach, accessibility of and engagement with the intervention across different socio-economic and ethnic groups. Descriptive statistics will be reported for the end-of-session questionnaires (capturing experiences of sessions including satisfaction, understanding of behavioural strategies, social identification) and fidelity to content checklists.

### Process evaluation

An approach similar to that described of the RCT will be taken. Thematic analysis of interview transcripts will be undertaken for each interview group (patients and facilitators). The Framework approach to index, sort, review and display data for both cross-case and within-case analyses will be used.

### Data management

Data management for the implementation study follows the same principles and practice as described for the RCT.

### Implementation study schedule

The implementation study schedule is equivalent to the PROGROUP arm in table 1, with the only differences being the omission of randomisation and 12 month timepoint.

## Ethics and dissemination

### Ethics approval and consent to participate

This study, including the adjusted programme of work, has been approved by West of Scotland REC3 (REC reference: 23/WS/0101).

### Data

Participants’ anonymity will be maintained through protective and secure handling and storage of patient information.

After the research programme has reported, the individual participant data that underlie the results will be available on request to the Chief Investigator and Sponsor, along with supplementary files as required.

### Dissemination policy

On completion of the research programme, the findings will be submitted to a peer-reviewed medical journal as open access. Lay summaries of the trial results will be made available to participants.

### Monitoring

The PROGROUP Programme Steering Committee has formally agreed to adopt the role of TSC for this trial. The TSC will meet every 6–7 months in accordance with an agreed set of terms of reference to review the progress of the trial and any SAEs and will report to the Sponsor. A Data Monitoring and Ethics Committee will not be convened for this trial, which is considered to pose low risk of harm to participants. The TMG meets monthly to review trial progress and to ensure appropriate management of the trial, in accordance with the terms of reference for the group.

## Discussion

Group-based behavioural interventions have been shown to be successful in supporting treatment of long-term conditions. With the increasing prevalence of obesity and severe obesity in the UK, there is a need to identify the optimum content, delivery modality, effectiveness and cost-effectiveness of the core behavioural intervention used in SWMS. Despite several studies showing benefits of a group-based intervention, there is an underrepresentation of people with severe obesity in the literature. As such, the PROGROUP programme aims to establish a new group-based intervention designed to meet these requirements for SWMS.

The RCT element of this study aims to compare the group-based weight-loss behavioural intervention, PROGROUP, with common current UC at SWMS. Following the approval by NICE in 2023 for the use of semaglutide,^63^ and in 2024 for the use of Tirzepatide,^64^ there have been mounting expectations, along with considerable uncertainty, about the likely availability of new obesity management medication in the NHS. As a result, recruitment to the RCT dropped below the rate originally predicted and required to achieve the original statistically powered sample size within the planned recruitment window. As such, this RCT will now compare PROGROUP and UC with descriptive statistics, cost-consequence analysis and parallel process evaluation. With the change in final data collection from 12 to 6 months post-randomisation, opportunities to continue later follow-up of participants outside of the programme timeline will be explored. In view of recently planned NHS changes that will provide for delivery of OMM in primary care settings,^65^ an implementation study has also been added to this work to assess the effectiveness of the PROGROUP intervention in primary care. The aim of this is to determine whether the intervention can be successfully implemented in a setting where the facilitators have not been formally trained in delivering behavioural weight-management interventions prior to this study. Implementation in primary care has the potential to open PROGROUP to a far larger group of people, as it will be open to those who have not had referral to SWMS. Analysis of the implementation study will therefore include a comparison of recruitment rates and interest in the programme with that in SWMS within the RCT. Thus, the study design, as originally planned, has been adapted in recognition of current and continuing changes in real-world weight management, and addresses the research questions in their current and evolving context.

## Administration

### Registration

The study was prospectively registered on the ISRCTN registry, reference number: ISRCTN13721429.

## Supplementary material

10.1136/bmjopen-2025-115791 online supplemental file 1
