# Supplementary material for: Evaluation of a group-based behavioural intervention (PROGROUP) versus usual care for weight management in adults with severe obesity: protocol for a randomised controlled trial with cost-consequence analysis and primary care implementation study
Source: BMJ Open. 2026 Jul 24;16(7):e115791. doi: 10.1136/bmjopen-2025-115791 (PMC13404495; doi:10.1136/bmjopen-2025-115791)
Supplement: online supplemental file 1 [file bmjopen-16-7-s001.pdf]

Title of Project: PROGROUP Trial IRAS ID: 322662

Principle Investigator: <PI> **Participant Study ID:** | | | | | |

PARTICIPANT CONSENT FORM (face-to-face)

Please initial each box*

1. I confirm that I have read the Information Sheet dated................... (version...........) for the above study. I have had the opportunity to consider the information, ask questions and have had these answered satisfactorily. ☐

2. I understand that my participation is voluntary and that I am free to withdraw at any time without giving any reason, without my medical care or legal rights being affected. ☐

3. I understand that if I am placed in the PROGROUP programme I will be expected to try to attend as many sessions as possible. ☐

4. I understand that I will be expected to attend a weight management service clinic at 6 months. ☐

5. I understand that if the study needs to be audited, relevant sections of my medical notes and data collected during the study may be looked at by individuals from the Sponsor organisation (University Hospitals Plymouth NHS Trust), the Peninsula Clinical Trials Unit (University of Plymouth) and regulatory authorities. I give permission for these individuals to have access to my records for this purpose. ☐

6. I give permission for the Peninsula Clinical Trials Unit (University of Plymouth) to store my name and contact details for the purpose of the study. ☐

7. I understand that the information collected about me will be used to support other research in the future and may be shared anonymously with other researchers. ☐

8. I understand that information about me that is held and maintained by the Tier 3 Weight Management Service may be used to contact me or provide information about my health status, for the purpose of this study only. ☐

9. I agree to my General Practitioner (GP) being informed of my participation in the study. ☐

10. I agree to take part in the PROGROUP Trial. ☐

Optional

Please initial each box*

11. I agree to provide my NHS number, so that the information I provide in this study may be combined with information held about me by other health or government organisations such as NHS England. ☐

12. I agree to provide a blood sample at baseline and 6m follow-up, for the purpose of the PROGROUP research, if my service does not collect these as part of my routine care. I understand that my local researcher will arrange an appointment to collect these samples. ☐

Please note, saying yes to any of the questions that follow does <u>not</u> commit you to anything but enables us to contact you in due course.

Please initial one box per question

13. We are hoping to conduct some research with a sample of participants to explore participants' experiences of taking part in the study. Are you interested in taking part in a short interview with a researcher by telephone or video call (e.g. Zoom)?

    Yes, I am happy to be contacted to discuss my experiences of the study with a researcher. I understand that my contact details will be passed to the researcher if required. ☐

    No, I do not want to be contacted about this. ☐

14. We may want to contact you at some point after this study has finished in order to assess your ongoing progress with weight management and related issues. Are you willing to be contacted for this purpose?

    Yes, I am happy to be contacted by a researcher after the study has finished to discuss my ongoing progress. I understand that my contact details will be passed to the researcher if required. ☐

    No, I do not want to be contacted. ☐

15. We may want to contact you about participation in future research opportunities. Are you willing to be contacted for this purpose?

    Yes, I am happy to be contacted by a researcher in relation to future research opportunities. ☐

    No, I do not want to be contacted. ☐

***The participant must initial each box on the consent form.**

______________________	______________________	____________
Print Name (Participant)	Signature	Date
Print Name (Person taking consent)	Signature	Date

PARTICIPANT CONSENT FORM (telephone)

***The person taking telephone consent must initial each box on the consent form.**

For consent taken over the telephone by a member of <site name> staff

I, name of person taking consent have read this consent form to participant name in full on dd / mm / /yyyy I confirm that they have:

- understood the participant information sheet and informed consent form *and*
- verbally agreed to the numbered points 1 to 10 *and*
- indicated their preferences for optional items 11 to 15.

Print Name (Person taking consent)	Signature	Date
