# Supplementary material for: Evaluation of a group-based behavioural intervention (PROGROUP) versus usual care for weight management in adults with severe obesity: protocol for a randomised controlled trial with cost-consequence analysis and primary care implementation study
Source: BMJ Open. 2026 Jul 24;16(7):e115791. doi: 10.1136/bmjopen-2025-115791 (PMC13404495; doi:10.1136/bmjopen-2025-115791)
Supplement: Supplementary file 2 [file bmjopen-2025-115791.reviewer_comments.pdf]

PEER REVIEW HISTORY

BMJ Open publishes all reviews undertaken for accepted manuscripts. Reviewers are asked to complete a checklist review form and are provided with free text boxes to elaborate on their assessment. These free text comments are reproduced below.

ARTICLE DETAILS

Title (Provisional)

An evaluation of a group-based behavioural intervention (PROGROUP) versus usual care for weight management in adults with severe obesity: Protocol for a randomised controlled trial with cost-consequence analysis and primary care implementation study.

Authors

Barnett, Anton; Hosking, Joanne; Tarrant, Mark; Creanor, Siobhan; Spencer, Anne; Ingram, Wendy; Sanders, Jeanette; Swancutt, Dawn; Lloyd, Jenny; Sheaff, Rod; Dean, Sarah; Byng, Richard; Hawkins, Lily; Hollands, Laura; Moghadam, Shokraneh; Abraham, Hanna; Aspinall, Paigan; Baldrey, Sarah; Edwards-Smith, Liz; Evans, Lucy; Gill, Laura; Gott, Hannah; Hind, Sarah; O'kane, Mary; O'shaughnessy, Emma; Perry, Steve; Sun, Weiming; Taylor, Adrian; Wilding, John; Wilson, Lucy; Pinkney, Jonathan

---

VERSION 1 - REVIEW

**Reviewer**	**1**
**Name**	**Leaviss, Joanna**
**Affiliation**	**University of Sheffield , SCHARR**
**Date**	**19-Mar-2026**
**COI**	**None**

I appreciate that the authors have had to re-design their RCT in order to take account of the rollout of obesity management medication (OMM) in the NHS. Although the aims of the RCT have been amended, the authors will conduct sensitivity analyses to explore use of OMMs on outcomes.

The key area that requires more clarity is the usual care group. It sounds as though usual care will be a specific programme, but that this may differ depending on location and availability? How will the authors attempt to disentangle any effects of the PROGROUP intervention from features of the usual care programmes? Plans to exclude "deliberate, facilitated, group-based management of behaviour change" sounds straightforward but what is the method for defining this? Will all usual care data be lumped together as one? Might

some usual care interventions have more in common with the PROGROUP intervention than other usual care programmes? Will they be comparable with each other in terms of duration, intensity and delivery? This has knock on effects for the cost saving analysis - how will usual care interventions be costed?

Follow-up: Data will no longer be collected at 12 months, instead analysed at 6 months only. This is barely end of treatment and there needs to be more explanation as to why this is justified. In particular, concerns about OMMs relate to what happens after treatment ends (if behaviours haven't changed), and it seems sensible to also record what is happening after treatment ends over a longer period, for those who are and are not taking OMMs.

Ethics: Can the authors confirm that the implementation study is covered under the original ethics approval, as this is not clear from that section.

VERSION 1 - AUTHOR RESPONSE

Reviewer's comments

Usual Care

- *"It sounds as though usual care will be a specific programme, but that this may differ depending on location and availability? How will the authors attempt to disentangle any effects of the PROGROUP intervention from features of the usual care programmes?"*

Usual care is not a specific programme. However, NICE guidance at the time (NICE CGL189) and subsequent guidance on the management of obesity in adults strongly emphasises that weight management programmes should be multidisciplinary, encompassing a focus on supporting behaviours around diet, lifestyle and physical activity. Therefore, the inclusion criterion for sites to participate in the RCT was to meet these NICE-stipulated criteria – without specifically offering group-based behavioural change interventions. In this respect, usual care is one clear type of programme, and the commonest in existence at the time.

Our pre-trial work, including discussions with potential trial sites across the UK established variability in service delivery (which was well-known), from usual care programme structure and length, its content, intensity, and theoretical focus. A defining feature of the PROGROUP group intervention is its focus on the facilitated development of psychological connections (or shared social identity) amongst members of the group. The intervention logic model prioritises such development, and associated group processes, as underpinning behaviour change leading to improved health outcomes. Accordingly, any site which already offers a group programme which purposely facilitates the patient group in this way is excluded from the trial. Therefore, the risk of contamination between the intervention and control arms is limited to educational content (for example, if a participant in the PROGROUP arm shared materials with someone from usual care), aspects of which is commonly available to all. Contamination of the social identity mechanism is not plausible. However, we have developed a process for checking for any contamination through the planned process evaluation.

Contamination effects will be collected at the 6 month follow up (see final paragraph in the Process Evaluation section in the manuscript). Intervention participants are asked whether they have discussed and/or shared resources with other patients in the weight management service and control participants whether they heard/saw anything about the PROGROUP sessions from other patients/staff.

Facilitators were also asked whether they used aspects of PROGROUP when working with the usual care patients and the importance of preventing contamination is covered in the facilitator training. This has been added to the 'Process Evaluation' section of the manuscript.

- *"Plans to exclude "deliberate, facilitated, group-based management of behaviour change" sounds straightforward but what is the method for defining this?"*

Please see above. Site eligibility is established through conversations with service leads and care staff at sites, including review of usual care materials, prior to confirmation of involvement in the trial. We take a deliberately conservative approach with particular scrutiny of usual care programmes involving patient groups: any which have dedicated group activities beyond provision of didactic educational information, such as group discussions and activities, are excluded. The 'Usual care' subsection of 'Treatments' in the manuscript has been amended to include this precise definition.

- *"Will all usual care data be lumped together as one? Might some usual care interventions have more in common with the PROGROUP intervention than other usual care programmes? Will they be comparable with each other in terms of duration, intensity and delivery? This has knock on effects for the cost saving analysis - how will usual care interventions be costed?"*

For the primary statistical analysis, all types of usual care will be included. We have pre-specified in the analysis plan that if any sites are to offer group-based care (not meeting the PROGROUP definition above), a sub-group analysis will be undertaken where the effect of usual care type will be explored. This has been added to the "Sample size and statistical methods" section of the manuscript. Number of appointments offered and n (%) attended for both usual care and PROGROUP will be reported.

We have highlighted above our strategies to ensure differences between usual care and PROGROUP in the trial.

We discuss the variability in usual care across the UK in our response above.

As above, the cost of usual care is expected to vary between sites. The type (e.g. individual/group session, telephone contact) and length of these contacts, by anonymised patient ID, will be received as electronic downloads from the provider. The unit costs of these services will be calculated based on this data and length of contact.

Follow-up

- *"Data will no longer be collected at 12 months, instead analysed at 6 months only. This is barely end of treatment and there needs to be more explanation as to why this is justified. In particular, concerns about OMMs relate to what happens after treatment ends (if behaviours haven't changed), and it seems sensible to also record what is happening after treatment ends over a longer period, for those who are and are not taking OMMs."*

The reviewer correctly highlights the benefits of longer term follow and we reassure that we do aim to do this. Specifically, during the consent process, we request permission to contact participants in future for additional research/data collection outside of the Programme timeline. The majority of participants have provided this permission. The 'Discussion' section of the manuscript has been updated to include this.

The decision to no longer collect 12-month data is a pragmatic response to improve site-recruitment and was endorsed by the trial steering committee. This followed to delayed, and then slowed, recruitment to the study due to post-COVID and obesity management medication NHS-rollout. With this change, the focus of the analysis has shifted to a cost-consequences approach, with statistical analyses being descriptive only.

Ethics

- *"Can the authors confirm that the implementation study is covered under the original ethics approval, as this is not clear from that section."*

The adjusted programme of work (including the implementation study) was approved by the West of Scotland Research Ethics Committee 3, via Amendments to the protocol (REC reference 23/WS/0101). A revision has been made in the revised manuscript in the 'Ethics approval and consent to participate' section for clarity.

VERSION 2 - REVIEW

**Reviewer**	**1**
**Name**	**Leaviss, Joanna**
**Affiliation**	**University of Sheffield , SCHARR**
**Date**	**11-Jun-2026**
**COI**	

Thank you for clarifying these issues. There are just a couple of very minor typos in the revised strengths and limitations section. Good luck with the study!

VERSION 2 - AUTHOR RESPONSE

Reviewer's comments

- *"There are just a couple of very minor typos in the revised strengths and limitations section."*

This has been amended.
